# Declines in health literacy and health-related quality of life during the COVID-19 pandemic: a longitudinal study of the Japanese general population

**DOI:** 10.1186/s12889-021-12092-x

**Published:** 2021-11-27

**Authors:** Hirono Ishikawa, Mio Kato, Takahiro Kiuchi

**Affiliations:** 1 Graduate School of Public Health, Teikyo University, 2-11-1 Kaga, Itabashi-ku, Tokyo, 173-8605 Japan; 2 Department of Health Communication, Graduate School of Medicine, The University of Tokyo, 7-3-1 Hongo, Bunkyo-ku, Tokyo, 113-8655 Japan

**Keywords:** COVID-19, Health literacy, Health related quality of life, Longitudinal study

## Abstract

**Background:**

During the coronavirus disease 2019 (COVID-19) pandemic, the importance of health literacy (HL) was addressed by public health researchers. We longitudinally examined the changes in general HL and health-related quality of life (HRQOL) between immediately before the COVID-19 outbreak and 1 year later, and how general HL before the outbreak was related to changes in HRQOL in the Japanese general population.

**Methods:**

Among the Japanese residents aged 20–79 years who participated in our previous study in 2017, 826 were followed-up via self-administered questionnaires in January 2020 and February 2021, for the purposes of this study. The HRQOL was measured using the SF-8, a short version of the SF-36 Health Survey, and general HL was measured using the short form of the European Health Literacy Survey Questionnaire (HL-SF12) in the 2020 and 2021 surveys.

**Results:**

The physical and mental dimensions of HRQOL as well as general HL declined significantly from immediately before the COVID-19 outbreak to 1 year later (*p* = .010, *p* < .001 and *p* < .001, respectively). The decline in HRQOL, especially the mental dimension, was more significant among women. A lower economic status was also related to a greater decline in HRQOL (*p* = .026 for the physical dimension and *p* = .012 for the mental dimension). Higher general HL before the COVID-19 outbreak was associated with a lesser decline in HRQOL in both the physical and mental dimensions (*p* = .040 and *p* < .001, respectively) after controlling for possible confounding variables such as gender and economic status.

**Conclusions:**

Healthcare support is crucial for vulnerable populations during and after the pandemic. General HL may be important for attenuating the decline in HRQOL, by enabling effective use of health information and adaptive behaviors toward health threats. Further studies are needed to better understand the association between HL and HRQOL.

## Background

Since the outbreak of coronavirus disease 2019 (COVID-19), many countries have experienced large-scale societal changes that have had a profound impact on everyday life and behaviors. Governments have introduced substantial restrictions on people’s movements, including stay-at-home orders, limitations on gatherings, and the closure of non-essential workplaces. Although this was crucial for preventing the spread of COVID-19, such restrictions have led to severe economic downturns and job insecurity [1], which in turn had negative impacts on health and health-related behaviors.

Several large studies in the US and Europe reported a deterioration in mental health and health behaviors between the time before and during the COVID-19 pandemic [2–8]. Although the declines were observed across sociodemographic groups, some groups exhibited a greater decline in health, such as the young and women [2, 5, 7]. Also, declines in health-related quality of life (HRQOL) from before to during the pandemic have been reported among adolescents [9] and cancer patients [10]. In Japan, the prevalence of depressive symptoms increased among older adults [11], and physical activity decreased in the general adult population [12, 13]. A reduced level of physical activity is associated with depression and anxiety [14]. Therefore, COVID-19 not only directly affected health but also indirectly impacted HRQOL by mandating changes in social norms and daily life.

During the COVID-19 pandemic, the importance of health literacy (HL) was addressed by public health researchers [15–22]. HL is defined as “people’s knowledge, motivation and competences to access, understand, appraise, and apply health information in order to make judgments and take decisions in everyday life concerning healthcare, disease prevention and health promotion to maintain or improve quality of life during the life course [23].” Research concerning HL in the context of the COVID-19 pandemic is nascent, so there is insufficient information on the relationships of HL with health behaviors and outcomes [24]. However, several cross-sectional studies have reported that lower HL is associated with confusion regarding COVID-19 [25] and erroneous beliefs [26–28]. Moreover, and protective behaviors are less frequently adopted in those with lower HL [27, 28]. In a Japanese study, higher HL was associated with exercise during the COVID-19 state of emergency [29]. Furthermore, studies of healthcare workers have reported that higher HL protects against mental health problems and is associated with higher HRQOL [30, 31]. Additionally, a cross-sectional study of outpatients reported that higher HL was associated with a lower likelihood of depression and higher HRQOL [32], which moderated the negative impact of fear of COVID-19 on HRQOL [33]. However, no longitudinal study has examined the association of general HL before the COVID-19 pandemic with changes in health status during the pandemic among the general public.

In this study, we longitudinally examined the changes in HRQOL and general HL between 2020 (immediately before the COVID-19 outbreak) and 2021 (1 year later) and how general HL before the outbreak was related to changes in HRQOL in the Japanese general population.

## Methods

### Participants

**Fig. 1:**
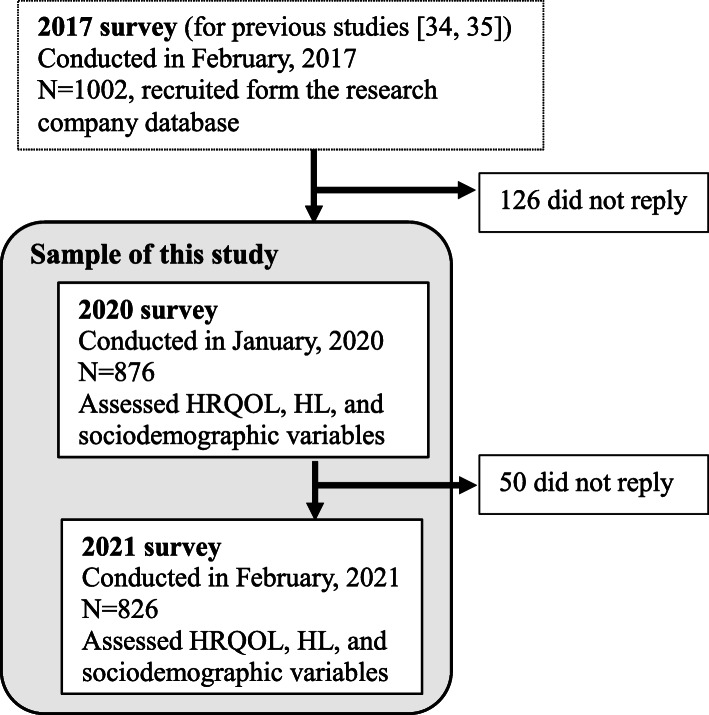
Timeline of the surveys and the sample of this study

Figure 1 shows the timeline of the surveys and the sample of this study. Participants were originally recruited for a survey in 2017 from a pool of Japanese residents obtained from a survey research company database. The survey was designed to investigate HL among the Japanese general population and its relationship with health-related behaviors [34, 35]. During the survey, we collected data from 1002 men and women aged 20–79 years. Respondents who met the inclusion criteria were randomly invited to participate by fax/mail. When enrolling participants from the database, we attempted to match them in terms of gender and age distributions with data from the 2016 national census of the Japanese population. Responses were obtained from potential participants until the target numbers for each gender and age group were met. Individuals who agreed to participate were asked to provide a completed consent form. A set of self-administered questionnaires was mailed to the participants. For the purpose of this study, the participants of the 2017 survey (*N* = 1002) were followed up using surveys in early January 2020 (*N* = 876) and again in February 2021 (*N* = 826). The first case of COVID-19 in Japan was confirmed on January 16, 2020. For this study, we used the data of 826 individuals who participated in both the 2020 and 2021 surveys. The response rate for the 2020 survey was 87.4% (876 of 1002), and that for the 2021 survey was 94.3% (826 of 876).

### Measures

#### Health-related quality of life

The SF-8, a short version of the SF-36 Health Survey, was used to measure HRQOL in the 2020 and 2021 surveys [36]. The SF-8 consists of eight items assessing general health, physical functioning, role limitations due to physical functioning, bodily pain, vitality, social functioning, mental health, and role limitations due to emotional functioning. Two summary scores, the physical component summary (PCS) and mental component summary (MCS) scores, are calculated as the weighted sum of each item’s score, transformed into T-scores, and normalized to the Japanese general population (50 points represents the national average value). The PCS and MCS scores indicate physical and mental functioning, respectively, with higher scores indicating a higher HRQOL.

#### Health literacy

The short form of the European Health Literacy Survey Questionnaire (HL-SF12) was used in this study. The original European Health Literacy Survey Questionnaire (HLS-EU-Q47) was developed to measure HL in the general population [37] based on a conceptual framework reflecting four information-processing dimensions (i.e., accessing, understanding, appraising, and applying) within three health domains (i.e., health care, disease prevention, and health promotion) [23], and has been validated in a Japanese population [38]. The HL-SF12 consists of 12 items selected from each domain of the HLS-EU-Q47 and has been validated in an Asian general population [39]. Each item assesses the perceived difficulty of completing a specific health-related task, rated on a four-point Likert scale (1 = very difficult, 2 = difficult, 3 = easy, and 4 = very easy) with a “don’t know” option. Responses of “don’t know” were treated as missing and not included in the calculations of the participants’ index scores [38]. Using the scores of all 12 items, we constructed a comprehensive general index for HL. Following the original study, a mean-based item raw score was computed for respondents who provided valid responses to at least 80% of the HL questions [40]. The index score was standardized to unified metrics from 0 to 50 using the following formula: (mean - 1) × (50/3). Cronbach’s alpha for the scale was 0.891 in 2020 and 0.889 in 2021.

#### Sociodemographic data

The following demographic data were obtained as part of both 2020 and 2021 survey: age (years), gender (male or female), educational attainment (junior high school, high school/vocational school, 2-year college, university or higher), self-assessed economic status (rated on a 10-point scale ranging from 1 = lowest to 10 = highest in society), work status (self-employed, full-time employed, part-time employed, and others [including retirees, homemakers, students, and individuals not in a paid job for any reason]), and the presence of a currently treated disease (i.e. whether participants had a disease being treated during the previous year [yes or no]).

### Statistical analysis

The changes in HRQOL and HL between 2020 and 2021 were examined using paired *t*-tests. The changes in HRQOL were calculated by subtracting the HRQOL scores of 2021 from those of 2020. Multiple linear regression analysis controlling for sociodemographic variables and HRQOL in 2020 was performed to examine the relationship between HL in 2020 and changes in HRQOL. The sociodemographic data obtained from the 2021 survey were used because these data were considered to reflect the situation during the first year of the pandemic. In addition to age, gender, and educational attainment, which were typically adjusted for in previous HL studies, other sociodemographic variables correlated with HRQOL or HL in the bivariate analysis (*p* < .20) were included in the regression models. Stratified analyses by gender were also performed. Missing data were deleted list-wise. Data were analyzed using Stata ver. 16.1 (StataCorp, College Station, TX).

## Results

### Participants’ characteristics and descriptive statistics

Table 1 summarizes the sociodemographic characteristics of the participants in the 2020 and 2021 surveys. In the 2021 survey, the mean age was 54.6 years (standard deviation [SD] = 16.0 years), and 48.6% were males. Of the 826 participants, 40.3% had graduated from university, and 47.1% were full-time workers, including self-employed and full-time employed workers. There were 358 individuals (43.3%) who had a disease for which they had received treatment during the past year.

**Table 1:** The characteristics of the study participants

	2020		2021	
	N	%	N	%
Age				
20–29	80	9.7	67	8.1
30–39	110	13.3	109	13.2
40–49	140	17.0	126	15.3
50–59	178	21.6	187	22.6
60–69	151	18.3	146	17.7
70-	167	20.2	191	23.1
Mean (SD)	53.4	16.0	54.6	16.0
Gender				
Male	401	48.6	401	48.6
Female	425	51.5	425	51.5
Educational attainment				
Junior high school	30	3.6	30	3.6
High school/Vocational school	348	42.1	348	42.1
2-year college	115	13.9	115	13.9
University or higher	333	40.3	333	40.3
Self-assessed economic status				
Low:1–3	139	16.8	139	16.8
Middle low: 4–5	354	42.9	337	40.8
Middle high: 6–7	292	35.4	301	36.4
High: 8–10	39	4.7	45	5.5
missing	2	0.2	4	0.5
Work status				
Self-employed	78	9.4	84	10.2
Full-time employed	320	38.7	305	36.9
Part-time employed	148	17.9	154	18.6
Others	278	33.7	281	34.1
missing	2	0.2	2	0.2
Presence of a currently treated disease				
Yes	358	43.3	358	43.3
No	442	53.5	456	55.2
missing	26	3.2	12	1.5

### Changes in HRQOL and HL between 2020 and 2021

There were significant declines in both dimensions of HRQOL (PCS and MCS) and HL between 2020 and 2021 (Table 2). The mean HL score was 30.5 (SD = 8.8) in 2020, which declined to 28.7 (SD = 8.8) in 2021. Although there are no normative data for this scale in the general Japanese population, the mean score for our 2017 survey using the 47-item version of the scale was 29.79 (SD = 7.41) [34], which was somewhat higher than the 25.3 (SD = 8.2) reported in a previous online survey study on the general Japanese population using the same scale [38], but still lower than the value of 33.78 (SD = 7.95) reported by a previous European study [40].

**Table 2:** Changes in HRQOL and HL between 2020 and 2021

		2020		2021		Change 2021–2020		
	N	Mean	SD	Mean	SD	Mean	SD	*p*-value^1)^
**HRQOL-physical**	817	44.83	7.13	44.15	7.38	−0.68	7.58	0.010
male	399	45.18	6.75	44.62	6.89	−0.56	6.94	0.107
female	418	44.50	7.48	43.70	7.80	− 0.80	8.15	0.045
**HRQOL-mental**	817	49.72	7.14	48.63	7.84	−1.09	7.49	<.001
male	399	49.82	7.08	49.33	7.53	−0.48	7.06	0.172
female	418	49.62	7.21	47.96	8.08	−1.66	7.84	<.001
**Health literacy**	730	30.53	8.80	28.75	8.82	−1.78	6.62	<.001
male	351	29.85	9.08	28.03	9.09	−1.83	6.71	<.001
female	379	31.16	8.49	29.41	8.52	−1.74	6.55	<.001

^1)^ Paired t-test for changes between 2020 and 2021

When stratified by gender, the declines in the PCS and MCS scores were statistically significant only among females. By contrast, HL declined significantly for both men and women.

### Factors related to the decline in HRQOL

As shown in Table 3, those with higher HL in 2020 had a significantly smaller decline in score for the physical dimension of HRQOL (PCS) than those with lower HL. A lower economic status and the presence of a disease under treatment were associated with a greater decline. When stratified by gender, HL in 2020 had a significant association with the PCS score only among women.

**Table 3:** Factors related to the decline in HRQOL-physical by gender

	Total (*N* = 749)		Male (*N* = 363)		Female (*N* = 386)	
	B	95% CI	B	95% CI	B	95% CI
Age	0.006	(−0.029 to 0.042)	0.032	(−0.016 to 0.079)	−0.024	(− 0.078 to 0.030)
Gender	−0.392	(− 1.466 to 0.683)				
Education	−0.109	(−0.607 to 0.389)	0.084	(−0.533 to 0.700)	− 0.457	(−1.282 to 0.368)
Economic status	**0.358**	(**0.043 to 0.672**)	0.164	(−0.245 to 0.573)	**0.527**	(**0.045 to 1.010**)
Having a disease	**−1.602**	(**−2.685 to − 0.520**)	**0.183**	(**−3.234 to − 0.372**)	−1.297	(−2.932 to 0.338)
Full-time worker	1.053	(− 0.102 to 2.209)	**1.665**	(**0.124** **to** **3.206**)	0.319	(−1.436 to 2.075)
HRQOL-physical at 2020	**−0.594**	(**− 0.663 to − 0.524**)	**0.564**	(**− 0.661 to − 0.466**)	**−0.616**	(**− 0.716 to − 0.515**)
HL at 2020	**0.057**	(**0.003 to 0.112**)	0.028	(−0.042 to 0.098)	**0.092**	(**0.007 to 0.177**)
(constant)	23.327	(18.448 to 28.205)	21.102	(15.231 to 26.972)	24.155	(17.707 to 30.603)
Adjusted R-squared	0.270		0.259		0.278	

Multiple linear regression analysis was used. Significant differences are printed in bold (*p* < 0.05)

Similarly, HL in 2020 was significantly associated with a decline in the score for the mental dimension of HRQOL (MCS) (Table 4). The decline was greater for women, those of lower economic status, and those with a disease under treatment. When stratified by gender, the relationship between HL in 2020 and a decline in MCS score was more evident among men, and working full-time was related to a greater decline in the MCS score among women.

**Table 4:** Factors related to the decline in HRQOL-mental by gender

	Total (*N* = 749)		Male (*N* = 363)		Female (*N* = 386)	
	B	95% CI	B	95% CI	B	95% CI
Age	0.019	(− 0.018 to 0.056)	0.017	(− 0.033 to 0.067)	0.027	(−0.028 to 0.081)
Gender	**−2.046**	(**−3.145 to − 0.947**)				
Education	−0.246	(−0.756 to 0.263)	− 0.535	(−1.179 to 0.108)	0.136	(− 0.692 to 0.965)
Economic status	**0.416**	(**0.093 to 0.738**)	0.409	(−0.019 to 0.837)	0.377	(− 0.111 to 0.865)
Having a disease	**−1.123**	(**−2.204 to − 0.042**)	− 0.786	(− 2.258 to 0.686)	−1.375	(− 2.965 to 0.215)
Full-time worker	− 0.916	(− 2.099 to 0.267)	0.310	(− 1.294 to 1.914)	**−2.014**	(**− 3.783 to − 0.246**)
HRQOL-mental at 2020	**− 0.502**	(**− 0.573 to − 0.431**)	**−0.486**	(**− 0.583 to − 0.388**)	**−0.520**	(**− 0.624 to − 0.417**)
HL at 2020	**0.104**	(**0.048 to 0.161**)	**0.136**	(**0.063 to 0.210**)	0.076	(−0.011 to 0.162)
(constant)	22.389	(17.543 to 27.234)	18.601	(12.791 to 24.412)	19.260	(12.885 to 25.634)
Adjusted R-squared	0.208		0.210		0.194	

Multiple linear regression analysis was used. Significant differences are printed in bold (*p* < 0.05)

## Discussion

This study longitudinally examined the changes in HRQOL and general HL between immediately before the COVID-19 outbreak and 1 year later, and how HL before the outbreak was related to changes in HRQOL in the Japanese general population.

Overall, scores for the physical and mental dimensions of HRQOL declined significantly from immediately before the COVID-19 outbreak to 1 year later. The findings are generally consistent with previous studies in Western countries that reported deterioration in mental health and well-being [2, 4–7]. A previous study also reported that loneliness was associated with a higher incidence of suicidal ideation during the COVID-19 pandemic [41]. In Japan, unlike some US states and European countries, strong legal restrictions such as a complete “lockdown” have not been enacted thus far. Instead, the government repeatedly declared a state of emergency and imposed a voluntary stay-at-home order, except for essential tasks. Nevertheless, previous studies on the Japanese population have reported increased mental health problems [11, 42] and decreased physical activity and perceived physical fitness [12, 13]. Our findings are in line with these reports.

Further, the decline in HRQOL, especially the mental dimension, was more significant among women, consistent with previous reports [2, 5, 7]. Women may experience a disproportional burden of the economic shock associated with COVID-19, greater increases in childcare responsibilities and interruptions to paid work, and more job loss [5]. Although the COVID-19 pandemic’s effects on employment have been less severe in Japan compared to Western countries, the employment of non-regular employees and young people has declined [43]. Female employment is suggested to be affected more than male employment, because women make up a large proportion of employment in industries involving interpersonal services, such as the food service and accommodation sectors, and many of those are engaged in non-regular employment [44]. In addition, among women, working full-time was associated with a greater decline in the score for the mental dimension of HRQOL. This may reflect difficulties in balancing work and family resulting from school closures and remote work during the pandemic. Mental health support for vulnerable populations such as women and those with a lower economic status is thus crucial during and after the pandemic.

Notably, the decline in HRQOL was greater in those with a disease currently being treated, possibly due to the challenges associated with managing a chronic condition during the pandemic. People with certain underlying medical conditions are at increased risk of serious illness from COVID-19. Previous studies reported that patients with chronic diseases reduced their number of medical visits during the pandemic, partly because of the fear of getting infected with COVID-19 at medical institutions [45, 46]. To reduce the increase in non-COVID-19-related morbidity and mortality, it is critical to encourage patients with chronic diseases to continue to receive care, and to develop healthcare services to support them, including those delivered by telephone or online.

General HL also declined significantly. HL is mediated by organizational structures and the availability of resources that enable people to access, understand, appraise, and use information and services in ways that promote and maintain good health for themselves and those around them [47]. In particular, the measure of HL used in this study is designed to measure the subjective manageability of health-related tasks, focusing on both individuals and underlying circumstances in which health-related tasks are performed [40, 48]. The lower score in 2021 may reflect difficulties in obtaining and understanding adequate information in the context of COVID-19, where many problems in health communication has emerged. During the pandemic, false or contradictory information spread rapidly via social media and other Internet outlets, and the “infodemic” (the global epidemic of misinformation) has posed a serious problem for public health [49]. Although governments and health authorities have provided information on the risk of COVID-19 and how to prevent contracting or spreading the infection, there has been concern that educational materials and websites on COVID-19 provide information at a readability level far exceeding that recommended for patient information [50, 51].

Higher general HL before the COVID-19 outbreak was associated with less decline in HRQOL. This is consistent with previous cross-sectional studies suggesting that higher HL was associated with a lower likelihood of mental problems and higher HRQOL [30–32]. As discussed above, HL is considered to play an important role in the acquisition, understanding, and use of information, which might have prompted the adoption of adaptive behaviors toward health threats [25–28], and moderated the negative impact of fear of COVID-19 on HRQOL [33]. In addition, as to the physical dimension, those with higher HL might have engaged in more health-promoting activities during the pandemic to maintain physical fitness [29]. Our findings indicate that higher HL before the pandemic might have protected against a decline in HRQOL after controlling for sociodemographic variables such as age, gender, and income. HL is personal knowledge and competencies that accumulate through daily activities, social interactions, and across generations [47]. Thus, HL is likely to be improved by education unlike other sociodemographic factors such as gender and economic status. Educational interventions at ordinary times to enable people to develop transferable skills in accessing, understanding, analyzing, and applying health information may be important to reduce health disparities during times of health risk.

This study had several limitations. First, the participants were recruited from the database of a survey research company; therefore, we were unable to include individuals uninterested in participating in such commercial surveys. However, the database made possible a longitudinal survey with comparatively few dropouts (5.7% between 2020 and 2021). Second, the sample may not be representative of the general population of Japan. The proportion of university graduates in the sample was approximately 40%, compared with 25% based on the 2017 Employment Status Survey by the Statistics Bureau of Japan. The generalizability of our findings should be carefully considered based on these characteristics. Third, self-administration of questionnaires requires at least a basic level of literacy, which may have biased the findings to some degree. Fourth, HL was measured using a self-report questionnaire. The responses represented the participants’ own perceptions and may have been different from the objective ability. Fifth, we did not know whether there were participants who had become infected with COVID-19, quarantined, or had close contact with infected persons. A previous study reported lower HRQOL among people with COVID-19 symptoms [32]. Also, health-related behaviors and mental health issues can affect HRQOL, but this was not examined in this study. Further studies taking account of these factors are needed to better understand the association between HL and HRQOL.

## Conclusions

In conclusion, the physical and mental dimensions of HRQOL and general HL declined significantly from immediately before the COVID-19 outbreak to 1 year later. The decline in HRQOL was more significant in those with lower economic status and a disease currently being treated, as well as among women, particularly for the mental dimension. Healthcare support for these vulnerable groups is crucial during and after the pandemic. Higher general HL before the COVID-19 outbreak was associated with a lesser decline in HRQOL. General HL may be important for attenuating the decline in HRQOL by promoting effective use of health information and adaptive behaviors toward health threats. Further studies are needed to better understand the association between HL and HRQOL.

## Data Availability

The datasets used and/or analysed during the current study are available from the corresponding author on reasonable request.

## Abbreviations

HL: Health literacy
HRQOL: Health-related quality of life
